# Ebstein Anomaly With QRS Fragmentation on Electrocardiogram

**DOI:** 10.1177/2324709616688710

**Published:** 2017-01-01

**Authors:** Prakash Acharya, Jonathan Ross Ang, Bernard Gitler

**Affiliations:** 1 Montefiore New Rochelle Hospital, New Rochelle, NY, USA; 2 University of Missouri Health Care, Columbia, MO, USA

**Keywords:** Ebstein anomaly, QRS fragmentation, electrocardiogram, paradoxical embolization

## Abstract

Ebstein anomaly is a rare congenital disorder that involves the tricuspid valve and the right ventricle. It is associated with interatrial communication, which allows for paradoxical embolization causing unilateral blindness. Abnormal conduction through the atrialized right ventricle leads to QRS fragmentation on electrocardiogram. Its presence suggests a more severe abnormality and a higher risk of arrhythmia. The QRS fragmentation disappears after corrective surgery with resection of the atrialized right ventricle.

## Introduction

Ebstein anomaly is a rare congenital heart disease with prevalence of one in about 20,000 live births (Table 1).^1^ It is associated with downward displacement of tricuspid valve (TV) and functional integration of right ventricular inlet into the right atrium.^2^ Patients usually present with arrhythmia and, rarely, with manifestation of paradoxical embolization.

**Table 1.** Prevalence of Congenital Heart Disease (Metropolitan Atlanta, 1978-2005)^1^.

Type of Congenital Heart Disease	Prevalence per 10 000 Live Births
All congenital heart diseases	67.7
Ventricular septal defect	Muscular: 14.2
	Perimembranous: 7.7
Atrial septal defect—secundum	6.1
Tetralogy of Fallot	4.6
Pulmonary valve stenosis	4.1
Patent ductus arteriosus	3.6
Ebstein anomaly	0.5

We report the case of an adult with previously undiagnosed Ebstein anomaly presenting with unilateral loss of vision. He was noted to have characteristic electrocardiographic findings of Ebstein anomaly including QRS fragmentation.

## Case Presentation

A 56-year-old male with diabetes mellitus presented with sudden onset of unilateral vision loss described as a curtain falling over his right eye on the day before presentation. The visual loss was preceded by 24 hours of retro-orbital pain and a throbbing headache. He denied motor weakness, abnormal body movement, or loss of consciousness. There was no chest pain, shortness of breath, or palpitations. Vital signs were normal. Examination of his right eye revealed a fixed 3-mm pupil with absent direct light reflex but intact consensual reflex and a pale fundus with a cherry red spot but no hemorrhages. Left eye was normal. A holosystolic murmur was heard over the left parasternal area. Chest examination and rest of the neurological examination were normal. Hematology and chemistries were within normal limits.

**Figure 1.**
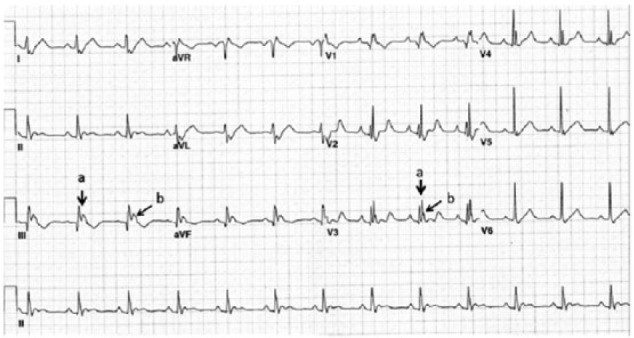
Initial ECG showing QRS fragmentation (a, fragmentation of QRS; b, broad positive deflection [R′] representing a second QRS).

Electrocardiogram (ECG) was performed (Figure 1). The ECG revealed normal sinus rhythm at a rate of 76 beats per minute with a right bundle branch block configuration, and borderline Q waves in leads III and aVF. The QRS complex was “fragmented” in leads I, II, III, aVL, aVF, and V1-V5. The QTc was prolonged at 490 ms. No prior ECG was available for comparison. On admission, this unusual ECG was erroneously interpreted as ST segment elevation indicative of inferior wall injury.

**Figure 2.**
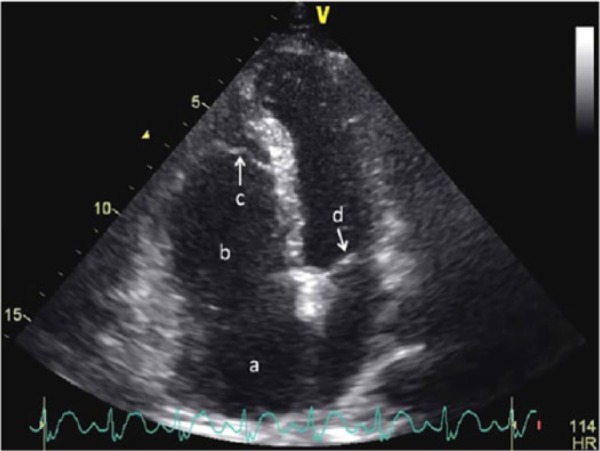
Echocardiography (apical 4-chamber view) showing features of Ebstein anomaly (a, right atrium; b, dilated atrialized portion of right ventricle; c, downward displacement of tricuspid valve; d, mitral valve).

Initial creatine kinase and troponin I tests were negative; erythrocyte sedimentation rate was normal. Computed tomography of the head was negative. The patient was started on aspirin, clopidogrel, and full-dose enoxaparin. Transthoracic echocardiography showed findings suggestive of Ebstein anomaly (Figure 2) with moderate tricuspid regurgitation (TR). Carotid Doppler and magnetic resonance angiography of the carotids were negative. As transthoracic echocardiography with bubble study showed the presence of an atrial septal defect, visual symptoms on admission was suspected to be secondary to a paradoxical embolus to the right eye.

**Figure 3.**
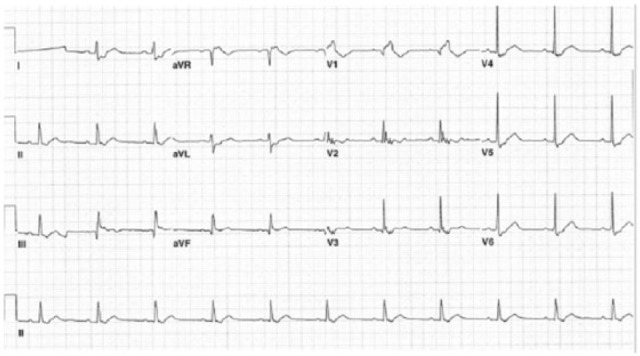
ECG after corrective surgery showing absence of QRS fragmentation.

No arrhythmia was noted on telemetry monitoring. Exercise nuclear stress test was done and it did not show myocardial ischemia. His vision improved considerably during his stay, and he was transferred for cardiac surgery. He eventually underwent TV replacement and atrial septal defect closure. QRS fragmentation was absent in the ECG performed after surgery (Figure 3).

## Discussion

**Figure 4.**
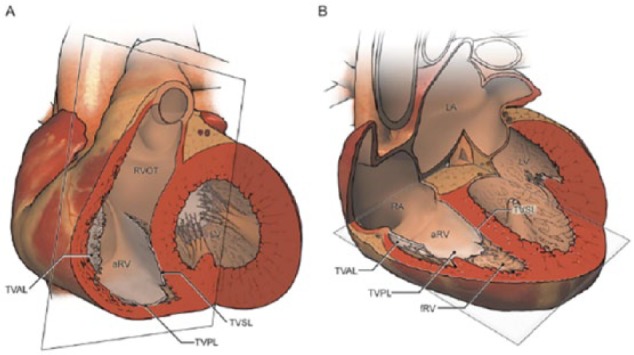
Three-dimensional reproduction of heart with Ebstein anomaly demonstrating characteristic findings of Ebstein anomaly in (A) short axis and (B) axial imaging. aRV, atrialized right ventricle; fRV, functional right ventricle; LA, left atrium; LV, left ventricle; RA, right atrium; TVAL, tricuspid valve anterior leaflet; TVPL, tricuspid valve posterior leaflet; TVSL, tricuspid valve septal leaflet. From Yalonetsky et al.3 Used with permission from Elsevier.).

Ebstein anomaly is characterized by adherence of septal and posterior leaflets of the TV leaflets to the underlying myocardium, malformation of the anterior leaflet, downward displacement of the functional annulus, dilation of the “atrialized” portion of the right ventricle (RV), and dilation of the right atrioventricular junction (Figure 4).^2,3^ The inlet portion of the RV gets functionally integrated with the right atrium forming the atrialized RV (aRV), which dilates disproportionately. An intra-atrial communication is found in over 80% of these patients.^2^

Clinical feature of Ebstein anomaly varies with the patient’s age. While infants and neonates present with cyanosis and heart failure, arrhythmia is the most common presentation in adults.^4^ About 30% patients have an anomalous pathway along the aRV, accounting for various arrhythmias including Wolff-Parkinson-White syndrome.^5^ There have been prior reports of paradoxical embolization in Ebstein anomaly leading to stroke.^6^

The common ECG findings in Ebstein anomaly include tall and broad P waves, prolonged PR interval, right bundle branch block pattern, fragmented QRS complex, and tachyarrhythmias due to accessory pathways usually present around the malformed TV.^2,7^ The fragmented QRS complex (“splintered,” “fractionated,” or “second QRS”), described as a normal shaped R wave directly followed by a broad positive deflection (R′) of lower amplitude, has been widely reported.^7-11^ Intracardiac mapping has shown that late depolarization in portions of the aRV correlates with the fragmented R′ of the ECG.^8^ These late positive low-voltage potentials (the R′) were found to disappear on signal averaged ECG in all patients after plication and resection of the aRV.^9^ Similarly, ECG performed after surgery in our patient shows the absence of QRS fragmentation (Figure 3).

Histological studies of the aRV have shown a decreased number of cardiomyocytes arranged in clusters within the fibrous network, completely replaced in some cases.^9,12^ Hence, it has been speculated that the enlarged right ventricle and progressive myocardial fibrosis and scarring within the aRV leads to the ECG finding of QRS prolongation and fragmentation.^10^ The fragmentation is usually noted in the inferior and precordial leads as the enlarged aRV occupies a greater portion of the inferior cardiac wall.^11,13^

The presence of a fragmented QRS is indicative of greater severity of Ebstein anomaly.^11^ These patients have larger aRV cavity volume, larger RV end diastolic volume, more severe TR, and worse RV systolic function.^10,11^ The comparatively larger aRV could indicate an increase in “arrhythmogenic substrate,” as the aRV has been shown to be irritable with the propensity for ventricular fibrillation.^8,11^ Hence, the presence of a fragmented QRS is independently associated with an increased incidence of ventricular arrhythmias.^11^ Increased atrial tachyarrhythmias are also noted in this group of patients, possibly secondary to structural changes, as these patients also have increased severity of right atrial enlargement and TR.^11^ The QRS duration tends to be longer in patients with QRS fragmentation than in those without. On long-term follow-up after corrective surgery, patients with prolonged QRS are noted to have increased mortality.^14^

In conclusion, adult patients with Ebstein anomaly can present with unilateral blindness secondary to paradoxical embolization. QRS fragmentation is a characteristic ECG finding that indicates a higher arrhythmia risk.
